# Selective enrichment, identification, and isolation of diclofenac, ibuprofen, and carbamazepine degrading bacteria from a groundwater biofilm

**DOI:** 10.1007/s11356-022-24975-6

**Published:** 2023-01-24

**Authors:** Márton Pápai, Tibor Benedek, András Táncsics, Till L. V. Bornemann, Julia Plewka, Alexander J. Probst, Daood Hussein, Gergely Maróti, Ofir Menashe, Balázs Kriszt

**Affiliations:** 1 Department of Molecular Ecology, Institute of Aquaculture and Environmental Safety, Hungarian University of Agriculture and Life Sciences, Páter K. U. 1, 2100 Gödöllő, Hungary; 2 Group for Environmental Metagenomics, Research Center One Health Ruhr of the University Alliance Ruhr, Faculty of Chemistry, University of Duisburg-Essen, Essen, Universitäts Str. 5, 45141 Essen, Germany; 3 Institute of Horticultural Sciences, Laboratories of Food Analysis, Hungarian University of Agriculture and Life Sciences, Páter K. U. 1, 2100, Gödöllő, Hungary; 4 Institute of Plant Biology, Biological Research Center, Temesvári Krt. 62., Szeged, Hungary; 5 Seqomics Biotechnology Ltd, Vállalkozók 7, 6782, Mórahalom, Hungary; 6 Water Industry Engineering Department, The Engineering Faculty, Kinneret Academic College On the Sea of Galilee, D.N. Emek Ha, 15132 Yarden, Israel; 7 BioCastle Water Technologies Ltd, Tzemah, Israel; 8 Department of Environmental Safety, Hungarian University of Agriculture and Life Sciences, Institute of Aquaculture and Environmental Safety, Páter K. U. 1, 2100 Gödöllő, Hungary

**Keywords:** Pharmaceuticals, Biodegradation, Metagenome binning, Nocardioides, Biofilm

## Abstract

Diclofenac, ibuprofen, and carbamazepine are three of the most widely detected and most concerning pharmaceutical residues in aquatic ecosystems. The aim of this study was to identify bacteria that may be involved in their degradation from a bacterial biofilm. Selective enrichment cultures in mineral salt solution containing pharmaceutical compounds as sole source of carbon and energy were set up, and population dynamics were monitored using shotgun metagenome sequencing. Bacterial genomes were reconstructed using genome-resolved metagenomics. Thirty bacterial isolates were obtained, identified at species level, and tested regarding pharmaceutical biodegradation at an initial concentration of 1.5 mg l^−1^. The results indicated that most probably diclofenac biodegrading cultures consisted of members of genera *Ferrovibrio*, *Hydrocarboniphaga*, *Zavarzinia*, and *Sphingopyxis*, while in ibuprofen biodegradation *Nocardioides* and *Starkeya*, and in carbamazepine biodegradation *Nocardioides*, *Pseudonocardia*, and *Sphingopyxis* might be involved. During the enrichments, compared to the initial state the percentage relative abundance of these genera increased up to three orders of magnitude. Except *Starkeya*, the genomes of these bacteria were reconstructed and annotated. Metabolic analyses of the annotated genomes indicated that these bacteria harbored genes associated with pharmaceutical biodegradation. *Stenotrophomonas humi* DIC_5 and *Rhizobium daejeonense* IBU_18 isolates eliminated diclofenac and ibuprofen during the tests in the presence of either glucose (3 g l^−1^) or in R2A broth. Higher than 90% concentration reduction was observed in the case of both compounds.

**Supplementary Information:**

The online version contains supplementary material available at 10.1007/s11356-022-24975-6.

## Introduction

In the last two decades, industrialized countries have witnessed an unprecedented increase in the consumption of pharmaceuticals due to the growing world population, aging societies, and increasing investment in the healthcare system. In 2001, the global pharmaceutical annual spending was $390 billion, which increased to $1.2 trillion in 2018, and is expected to grow to $1.5 trillion by 2023 (IQVIA 2019). Simultaneously with the increase in global pharmaceutical consumption, the appearance and concentration of pharmaceutically active compounds (PhACs) in the environment is expected to increase. PhACs are ubiquitous emerging pollutants and are one of the most concerned environmental problems nowadays (Patel et al. 2019; Khan et al. 2022a).

Numerous studies have reported the occurrence of PhACs in surface water, groundwater, and drinking water globally (Patel et al. 2019), in the European Union (EU, Loos et al. 2013), in the USA (Kostich et al. 2014), and in China (Liu and Wong 2013). The German Federal Environmental Agency reviewed the literature reporting data from environmental monitoring of pharmaceutical residues in 75 countries across the globe. In total, 178,708 data entries from 1519 publications have been reviewed (Eike et al. 2019). According to the survey, globally 771 pharmaceutically active substances or their transformation products have been detected, 596 for the EU and 269 for Germany. The vast majority of the detected PhACs or their residues have been reported in wastewater treatment plant (WWTP) effluents (globally 613, EU 474, and Germany 216). Globally, in surface water, groundwater and drinking water 528 substances have been detected. According to the study, 19 substances were detected in surface water, groundwater or drinking water in all five UN regions (Western Europe and Others Group, Latin American and Caribbean Group, Eastern Europe, Asian Group, and African Group; http://www.pharmaceuticals-in-the-environment.org; Eike et al. 2019). The most frequently detected pharmaceuticals in the Western European and Latin American and Caribbean groups were diclofenac (DIC), ibuprofen (IBU) and carbamazepine (CBZ).

DIC, IBU, and CBZ are the most commonly detected PhACs in freshwater ecosystems globally as well. They can be detected in surface and groundwater, as well as finished drinking water (Heberer 2002; Kleywegt et al. 2011; Lapworth et al. 2012; Hughes et al. 2013; Kozisek et al. 2013; Carmona et al. 2014; Luo et al. 2014; der Beek et al. 2016; Eike et al. 2019; Khan et al. 2021a, b). These compounds enter the environment through effluents of municipal wastewater treatment plants (Stuart et al. 2012; Santos et al. 2013; Petrović et al. 2014; Al-Tarawneh et al. 2015; Hapeshi et al. 2015; Chiffre et al. 2016; Paíga et al. 2016; Subedi et al. 2017), hospitals (Verlicchi et al. 2010; Santos et al. 2013; Tran et al. 2014; Khan et al. 2020a) and pharmaceutical industry (Ashfaq et al. 2017; Lin and Tsai 2009; Sim et al. 2011), from septic tanks (Subedi et al 2015), animal husbandry/veterinary medicine (especially DIC; Risebrough 2004; Shultz et al. 2004; Taggart et al. 2007), and from improper disposal of expired and unused drugs (Daughton and Ternes 1999; Heberer 2002; Paíga et al. 2013; Lu et al. 2016; Insani et al. 2020), etc.

PhACs are gaining attention since their residues can produce adverse effects on aquatic organisms (Jobling et al. 1998; Gross-Sorokin et al. 2006; Kidd et al. 2007; Loos et al. 2013; Küster and Adler 2014). The most alarming adverse effects of PhACs and residues can be demonstrated on the example of DIC. In South Asia, the extensive use of DIC in veterinary medicine led to the deaths of millions of vultures that fed on dead animal carcasses containing the drug, causing some populations to decline by more than 99% since the 1990s (Oaks et al. 2004; Shultz et al. 2004; Swan et al. 2006; Becker 2016). The appearance of PhACs in drinking water raises human health concerns too (Khan et al. 2022b). Although PhACs concentrations in drinking water are in the range of ng l^−1^ and are far lower than their therapeutic doses, long-term effects of such doses on the human health are still unknown (Kim et al. 2015). As it has been demonstrated by several studies, at environmentally relevant concentrations DIC, IBU, and CBZ, the target compounds of the present study, may have ecotoxicological impacts on rainbow trout (*Oncorhynchus mykiss*), mussels (*Mytilus galloprovincialis* and *Dreissena polymorpha*), crustacean (*Gammarus pulex*), adult Zebrafish (*Danio rerio*), Japanese medaka (*Oryzias latipes*), etc. (Schwaiger et al. 2004; Flippin et al. 2007; Triebskorn et al. 2007; Gonzalez-Rey and Bebianno 2014; Parolini et al. 2011; Mezzelani et al. 2016; Chopra and Kumar 2020).

From the above, it is obvious that conventional wastewater treatment facilities are inefficient in the elimination of PhACs which consequently accumulate in the environment and exert toxic effect on both aquatic and terrestrial ecosystems. Therefore, it must be a priority to better understand the spatial and temporal distribution of these compounds, as well as to determine their ultimate environmental fate. It is also essential to understand the microbiology laying behind PhAC biodegradation, and to uncover the nature’s inherent biodegradation capacity. The development of environmentally friendly, sustainable, and innovative biotechnological approaches for the elimination of these emerging pollutants from the environment is indispensable too. The new biotechnological approaches could be used as alternatives or efficiency-enhancing accessory units of conventional wastewater treatment plants. It has to be mentioned that over the years several technologies have been developed for removing PhACs from WWTP effluents (activated carbon, ion exchange, UV radiation, reverse osmosis, advanced oxidation processes, photocatalysis, membrane filtration, etc.), but compared to biotechnological methods these techniques are expensive, have a large ecological footprint and are not always efficient (Sui et al. 2010; Bolong et al. 2009; Aguinaco et al. 2012; Bernabeu et al. 2012; Prieto-Rodriguez et al. 2012; Sousa et al. 2012; Grover et al. 2011; Kleywegt et al. 2011; Snyder et al. 2007; Huber et al. 2003; Khan et al. 2020a, 2020b, 2020c; Khan et al. 2022c). Nevertheless, since the physicochemical and biological approaches are designed to remove different concentration ranges, the combination of these techniques may be the most efficient.

The goal of the present study was to selectively enrich, identify and isolate potentially DIC, IBU, and CBZ degrading bacteria from a groundwater biofilm. These three compounds were targeted because they are the most widely detected PhACs in aquatic ecosystems showing elevated concentrations and ecotoxic properties. The studied biofilm developed on the surface of a stainless-steel submersible pump of a Pump & Treat system, treating gasoline contaminated groundwater (for more details regarding the sampling site and the treatment system see Benedek et al. 2016). As it was determined previously, biofilm samples collected from the Pump & Treat system proved to be the habitat of a complex, dynamic network of interconnected, mutually dependent, simple- (such as BTEX—benzene, toluene, ethyl-benzene, and xylenes) and polycyclic aromatic hydrocarbon (PAH) degrading chemoorganotrophic bacteria with representatives of more than 600 genera of 40 classes of bacteria. Key biodegradative functional genes involved in simple- and PAH degradation, such as catechol 2,3-dioxygenases (*C23O*) belonging to I.2.A, I.2.B and I.2.C subfamilies, benzyl succinate synthase (*bssA*), and naphthalene 1,2-dioxygenase (NDO), were detected in the biofilm bacterial community (Benedek et al. 2016, 2018, and 2020). We hypothesized that the biofilm with high phylogenetic and functional diversity harbors bacteria capable of degrading DIC, IBU, and CBZ, because of the similar aromatic ring structure of these compounds. It has to be added, that these aromatic compounds have aromatic rings with different properties in terms of their tendency to stabilize aromaticity which leads into different reactivity. We further assumed that the studied bacterial biofilm behaves as a model community for the environment. By examining the effect of the three most widespread PhACs on the biofilm and potential identification of DIC, IBU, and CBZ degrading autochthonous bacteria, it can be concluded whether the environment possesses an inherent ability to cope with pharmaceutical residues. To the best of our knowledge, no contamination with pharmaceutical residues has ever been encountered at the sampling site. Throughout the study, cultivation-dependent conventional microbiological approaches were combined with cultivation-independent genome-resolved metagenomics with the result of delivering high quality genomes of the community involved in PhACs degradation. Pharmaceutical degrading isolates obtained during this study will serve as a basis for future development of biotechnological approaches for the elimination of PhACs from aquatic ecosystems.

## Materials and methods

### Biofilm sampling

The biofilm sample was collected into sterile 50-ml tubes in December 2019 in the Central Region of Hungary from a Pump & Treat system (well BUT-1) treating gasoline contaminated groundwater (for more details about the system, please see Benedek et al. 2016). After sampling, the biofilm was stored on ice and processed immediately upon arrival to the laboratory.

### Selective enrichment and identification of potentially DIC, IBU, and CBZ degrading bacteria

Selective enrichment cultures containing either DIC, IBU, or CBZ (100 mg l^−1^) as their sole source of carbon and energy were started in mineral salts solution supplemented with vitamins (Fahy et al. 2006). Enrichments were done in 300 ml Erlenmeyer flasks (sealed with cotton wool) containing 100 ml of enrichment media inoculated with 1 ml of biofilm bacterial suspension (0.5 g biofilm suspended in 50 ml saline solution 0.9% NaCl). Enrichment cultures were incubated at 28 °C on a rotary shaker (150 rpm) for 1 month. After the first month 10 ml of each enrichment culture was transferred to 90 ml of fresh enrichment medium and incubated again for an additional month. The transfer of enrichment cultures was repeated once more. In total, the selective enrichment of potentially pharmaceutical degrading biofilm bacteria was done for 3 months.

During the enrichments, apart from the target compounds, no co-metabolic substrates were used.

### Community DNA extraction from the enrichments, metagenome studies

Extraction of the total community DNA from the initial biofilm sample (0.5 g) and the enrichment cultures of each month was carried out by using the DNeasy® PowerBiofilm Kit (Qiagen, Germany) following the instructions of the manufacturer. 40 ml of the enrichment cultures were centrifuged at 2360 g for 15 min using a Rotanta 460 R centrifuge (Hettich, Germany), and the community DNA was extracted from the pellet.

#### Metagenome studies of the enrichment cultures

To precisely determine the phylogenetic diversity of the enriched samples, as well as to assess temporal dynamics that occurred due to the selective enrichment, shotgun metagenome sequencing was performed on the total community DNA of the initial biofilm sample as well as on the enrichment cultures (3–3 samples for DIC, IBU, and CBZ) by using the Illumina platform (Illumina Inc., USA). The quantity of DNA samples was estimated using a NanoDrop ND-1000 spectrophotometer (NanoDrop Technologies, Wilmington, USA) and a Qubit 2.0 Fluorimeter (Life Technologies, Carlsbad, USA). DNA purity was tested by agarose gel electrophoresis and on the Agilent 2200 TapeStation (Agilent Technologies, Santa Clara, USA). Libraries for Illumina sequencing were prepared using the NEBNext Ultra II Library Prep Kit. The metagenome sequencing was performed using an Illumina with NextSeq platform with a NextSeq 500/550 High Output Kit v2.5. Galaxy Europe server was employed to pre-process the raw sequences (i.e., sequence filtering, mapping, quality checking). Low-quality reads were filtered by Prinseq (min. length: 100; min. score: 15; quality score threshold to trim positions: 20; sliding window used to calculated quality score: 1). Filtered sequences were checked with FastQC. The filtered sequences produced by Prinseq were assembled with Megahit (Li et al. 2015a, b) (minimum contig length: 2000; minimum k-mer size: 21; maximum k-mer size: 141). Downstream taxonomical analysis and visualization of the retained reads were performed by the MEGAN6 software (Huson et al. 2007). Raw metagenome sequence reads are available on NCBI under the following BioProject accession number PRJNA782474; the BioSample accession number of the initial biofilm is SAMN23424265 and of enrichment cultures are SAMN23439944—SAMN23439952.

#### Reconstruction of bacterial genomes from metagenomes

In the case of the 3-month-old enrichment cultures, deep-metagenome sequencing was performed on the community DNA of samples as described above; 3.6, 4.4, and 4.7 Gigabase pairs were obtained for DIC, IBU, and CBZ enrichments, respectively. Raw metagenome sequence reads are available on NCBI under the BioProject accession number PRJNA782474 and SRA accession numbers SRR20688922 (DIC), SRR20688921 (IBU) and SRR20688920 (CBZ). For the reconstruction of bacterial genomes from the obtained metagenomes, the following pipeline was used.

##### Assembly and processing of metagenomes

Quality-controlled reads were assembled using MetaSPAdes version 3.15.0 (Nurk et al. 2017). Prodigal (Hyatt et al. 2010) in meta mode was used to predict genes on scaffolds ≥ 1000 bp length. Genes were annotated using diamond blast [(Buchfink et al. 2015); E-value cutoff 10^−5^] against the UniREF100 database (The UniProt Consortium 2019). Scaffold coverage was calculated from mappings of metagenomic reads against assemblies with Bowtie2 (Langmead and Salzberg 2012) in sensitive mode and a consensus taxonomy was determined for each scaffold based on the taxonomic annotations of individual genes on respective scaffolds against the UniRef100 database as described in Bornemann et al. (2020).

##### Binning of genomes from metagenomes (MAGs)

Genomes were binned with two binners: Abawaca (https://github.com/CK7/abawaca) with either 3 kbp and 5 kbp or 5 kbp and 10 kbp as respective scaffold fragmentation minimum and maximum parameters, and MaxBin2 (Wu et al. 2016) with both marker sets. All generated bins were aggregated with DAS Tool (Sieber et al. 2018) and curated with uBin (Bornemann et al. 2020). The taxonomic origin of reconstructed genomes was determined using the GTDB-tk classify_wf workflow with GTDB version r89 (Chaumeil et al. 2020). Genomes were annotated within the Microbial Genome Annotation & Analysis Platform MicroScope (MaGe, Vallenet et al. 2020). Additionally, putative functions of genes associated in the metabolism of xenobiotics were identified and bioinformatically analyzed by using MaGe in conjunction with the UniProt database (http://www.uniprot.org/; The UniProt Consortium 2019).

### Bacterial isolations

The isolation of potentially pharmaceutical degrading bacteria was performed after each month of enrichment. One ml from each enrichment culture was used for the preparation of tenfold serial dilutions in physiological salt solution (0.9% NaCl). Serially diluted samples (100 µl) were spread onto the surface of R2A agar plates (proteose peptone 0.5 g, casamino acids 0.5 g, yeast extract 0.5 g, dextrose 0.5 g, soluble starch 0.5 g, dipotassium phosphate 0.3 g, magnesium sulfate·7H_2_O 0.05 g, sodium pyruvate 0.3 g, agar 15 g, pH 7). After inoculations, the Petri-dishes were incubated at 28 °C for 48 h. Developed colonies, showing different morphologies, were purified by streak plating technique and maintained on R2A agar slants at 4 °C, and stored long-term at − 80 °C in a glycerol-R2A solution (30% v/v).

#### 16S rRNA gene-based identification of isolates

As a first step, the genomic DNA of isolates was extracted by using the DNeasy® UltraClean® Microbial DNA isolation Kit (Qiagen, Germany) following the instructions of the manufacturer. Next, bacterial species specific *16S rRNA* genes were PCR amplified by using the universal bacterial primers 27F (5′-AGAGTTTGATC(A/C)TGGCTCAG-3′) and 1492R (5′-TACGG(C/T)TACCTTGTTACGAC TT-3′). The PCR reaction mixture in a final volume of 50 μl contained, 5 μl 10 × DreamTaq™ buffer (ThermoFisher Scientific, Lithuania) with MgCl_2_ (2 mM), 0.2 mM of each dNTP, 0.1 μM of each primer, 1 U of DreamTaq™ DNA Polymerase (ThermoFisher Scientific, Lithuania), ~ 30 ng extracted DNA and nuclease free water up to the final volume. The temperature profile used for the amplification was an initial annealing for 3 min at 95 °C followed by 32 cycles of denaturation for 30 s at 94 °C, annealing for 30 s at 52 °C, elongation for 1 min at 72 °C and then a final extension for 10 min and 10 s at 72 °C. All amplifications were analyzed under UV light after electrophoresis in 1% (w/v) agarose gel stained with ethidium-bromide. The PCR amplicons were purified by using NucleoSpin® Gel and PCR Clean-up set (Macherey–Nagel, Germany). Subsequently, *16S rRNA* gene nucleotide sequences were determined with Sanger-sequencing by using BigDye Terminator v3.1 Cycle Sequencing Kit (Life Technologies, USA); the sequences were analyzed with ABI 3130 Genetic Analyzer (Life Technologies, USA). The resulted sequences were edited and assembled using MEGA X (Kumar et al. 2018), then homology BLAST searches (Altschul et al. 1997) were made in the GenBank database (http://www.ncbi.nlm.nih.gov/BLAST/). EzTaxon-e server carried out the determination of the closest type strains of isolates on basis of *16S rRNA* genes (http://eztaxon-e.ezbiocloud.net/, Kim et al. 2012). *16S rRNA* gene sequence data obtained in this study was deposited in the GenBank (Table 2).

### Pharmaceutical degradation screens for isolates

#### The resazurin screening assay

Isolated bacterial strains were screened for their ability to grow in the presence of individual pharmaceutical compounds added to the test solutions as single sources of carbon (10 mg l^−1^, either DIC, IBU, or CBZ). Screenings were conducted in triplicates in 100 ml of Bushnell-Haas mineral medium (CaCl_2_ · 2H_2_O 0.002 g, MgSO_4_ · 7H_2_O 0.02 g, NH_4_NO_3_ 1 g, KH_2_PO_4_ 1 g, K_2_HPO_4_ 1 g, FeCl_3_ ∙ 6H_2_O 0.005 g, H_2_O 1 l, with pH 7) supplemented with resazurin redox indicator (1 mg l^−1^). The test solutions were inoculated with 100 µl of bacterial cell suspension (OD_600_ = 1) obtained in physiological saline. Abiotic test solutions, and blank samples containing the medium without a carbon source were also set up and used as controls. Activity of the tested isolates was measured spectrophotometrically by measuring the absorbance of the test solutions at 610 nm (absorbance of the resazurin indicator, A610). As compared to the control samples (abiotic control and blank samples), a lower A610 value of the inoculated and DIC, IBU or CBZ supplemented test solution indicated an activity of the tested isolate in the presence of the pharmaceutical compound, signaling a presumable biodegradation capability. The higher the microbial activity in the test solutions, the more intense is the decrease in A610 value. The bigger the difference in the absorbance value at 610 nm between the controls and the test solutions the higher is the metabolic activity of the tested isolate. Test runs were incubated for two weeks on a rotary shaker at 145 rpm and 27 °C. At the beginning of the experiment all microcosms (including abiotic and blank samples) showed almost the same A610 value of 0.146 ± 0.002.

According to Tizzard et al. (2006), resazurin-based assays can be used for rapid screening for degradative capabilities of bacteria. Moreover, the same approach was successfully used in our previous study for the screening of simple aromatic hydrocarbon degrading bacteria (Benedek et al. 2018).

#### HPLC measurements

In order to determine more precisely the pharmaceutical biodegradation capacity of those bacterial isolates that showed high activity in the pharmaceuticals amended resazurin test solutions, new microcosm experiments were set up; the concentration of pharmaceuticals was followed using HPLC as described earlier (Benedek et al. 2022). Briefly, the microcosm experiments were conducted in triplicates in 50 ml of Bushnell-Haas mineral medium amended with one of the investigated pharmaceutical compounds as sole source of carbon and energy (1.5 mg l^−1^). Co-metabolic biodegradation microcosms were also set up, next to the pharmaceutical compound easily assimilable carbon sources such as yeast extract (0.05 or 0.3 g l^−1^) or glucose (0.5 or 3 g l^−1^) were added. Biodegradation experiments were also carried out in R2A broth containing the same concentration of pharmaceuticals. The test solutions were inoculated with 50 µl of bacterial cell suspensions (OD_600_ = 1). Abiotic controls, containing medium without an inoculum were also set up. Test runs were incubated for 4 weeks on a rotary shaker at 145 rpm and 27 °C.

Prior to injection into the HPLC instrument the aqueous samples were filtered using Whatman cellulose acetate syringe filters (0.45 µm). Samples were measured on a Chromaster Hitachi instrument consisting of a Model 5110 pump, a Model 5210 autosampler, and a Model 5430 Diode-array detector (DAD). Data processing was done using EZChom Alite software. The separation of the pharmaceutical compounds was performed on Ascentis C18, 150 × 0.46 mm column with isocratic elution of 50:50% (v/v) 0.02 M KH_2_PO_4_-acetonitrile at a flow rate of 0.8 ml min^−1^. The DAD was operated at a wavelength range between 190 and 400 nm. For the quantitative determination, a calibration curve for each compound was generated between concentration and absorbance at maximum wavelengths (DIC 202 nm, IBU 196 nm, and CBZ 214 nm). The compounds were identified based on comparison of retention time and spectral characteristics with those of standard solutions.

## Results

### Population dynamics due to selective enrichments

For metagenome analysis of each sample, 1.2–4.5 million sequence reads were generated (Online Resource 1).

**Fig. 1:**
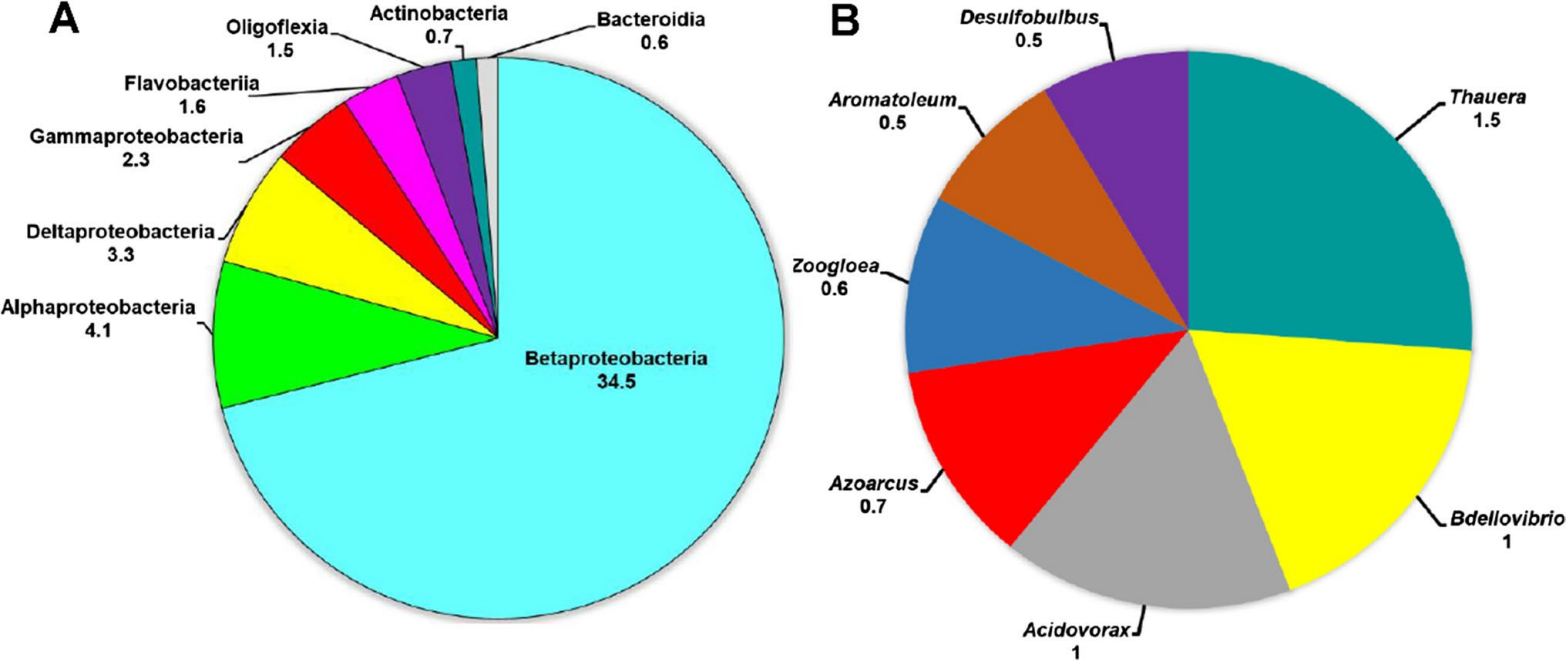
Taxonomic composition of the initial biofilm bacterial community at the class (**A**) and genus level (**B**) as assessed with shotgun metagenome sequencing. Numbers represent the relative abundance, only taxa with a percentage relative abundance  ≥  than 0.5% are shown

According to Fig. 1, the starting biofilm bacterial community at the class level was dominated overwhelmingly by Betaproteobacteria, followed by Alpha-, Delta-, and Gammaproteobacteria, as well as Flavobacteriia. At the genus level, representatives of the genera *Thauera*, *Bdellovibrio*, *Acidovorax*, *Azoarcus*, *Zoogloea* were the most abundant (percentage relative abundance ≥ 0.5%) (Fig. 1).

**Fig. 2:**
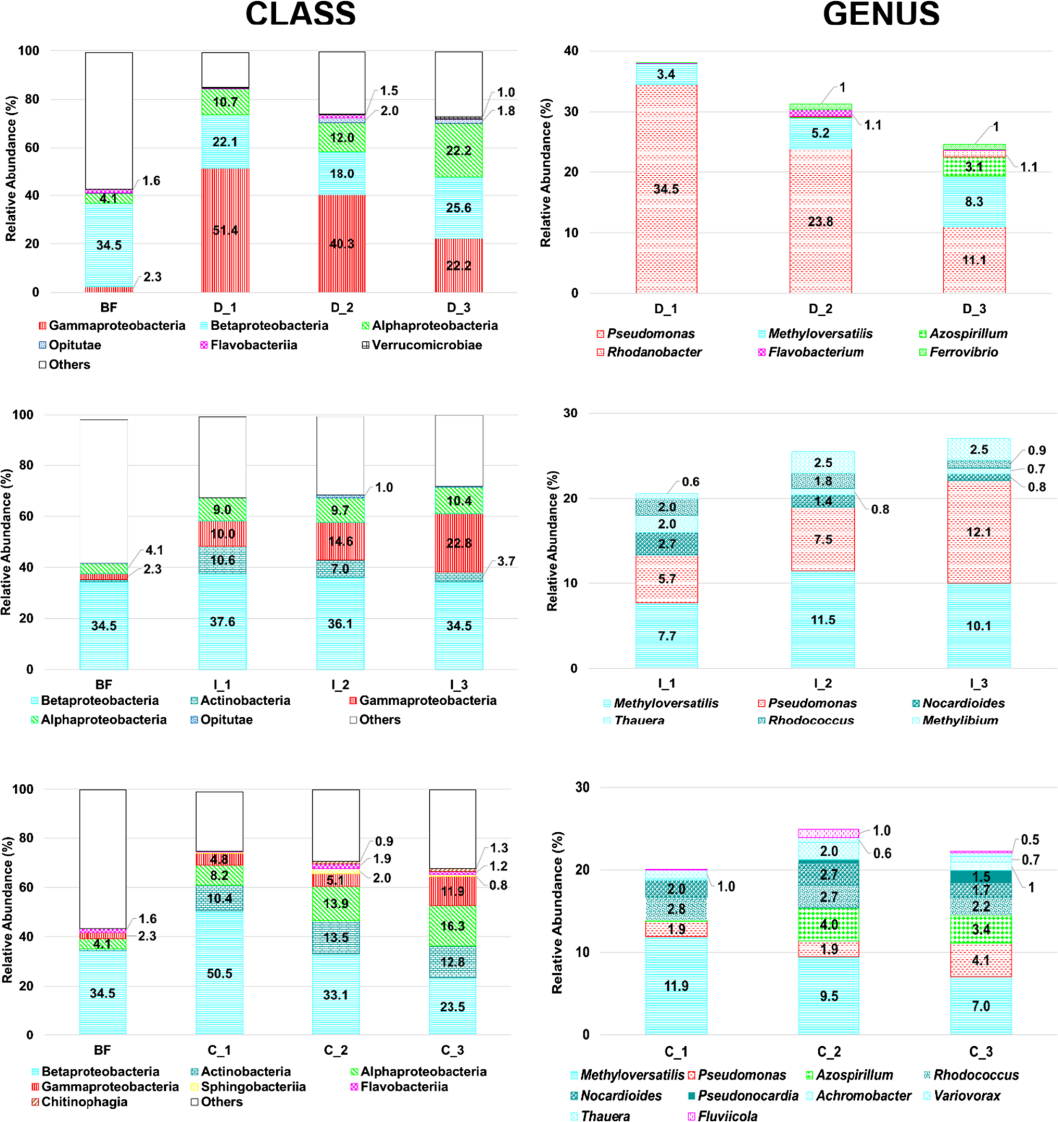
Taxonomic composition and temporal dynamics of bacteria found in the DIC (D_n), IBU (I_n), and CBZ (C_n) enrichment during the three-month long selective enrichment period (n), compared to the initial biofilm bacterial community (BF). Bacterial classes and genera with a percentage relative abundance ≥ 1% in at least one of the samples are shown only. Others represent the sum of phyla with a percentage relative abundance lower than 1%

All the DIC enrichments were dominated by Alpha-, Beta-, and Gammaproteobacteria. While in DIC enrichments, the Gammaproteobacteria were the most abundant, and the IBU and CBZ enrichments were dominated by Betaproteobacteria. In addition, Actinobacteria became a substantial member of the community with a percentage relative abundance between 4 and 14% only in IBU and CBZ enrichments (Fig. 2).

At genus level, the DIC enrichments were overwhelmingly dominated by *Pseudomonas* species followed by the genera *Methyloversatilis*, *Azospirillum*, *Ferrovibrio*, *Rhodanobacter*, and *Flavobacterium*. By contrast, *Methyloversatilis* species were the most dominant in the IBU and CBZ enrichments, whereas the genus *Pseudomonas* placed second/third based on percentage relative abundance. In the latter two enrichment types, representatives of the class Actinobacteria such as *Nocardioides*, *Rhodococcus*, and *Pseudonocardia* became key community members. In the case of the last 2 months of CBZ enrichment, the genus *Azospirillum* reached notable proportions too, reaching 3.4–4% in percentage relative abundance (Fig. 2).

**Fig. 3:**
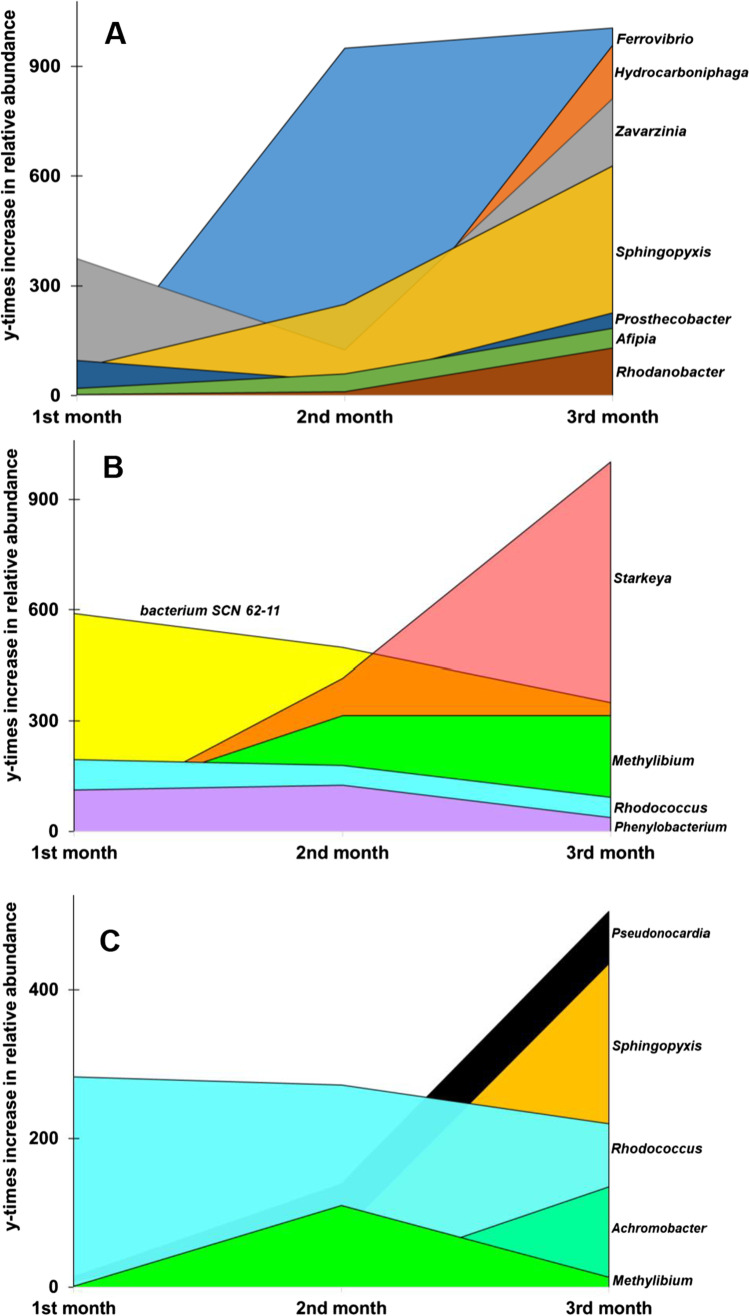
Temporal dynamics of bacterial genera with ≥ 0.5%, showing at least a 100-fold increase in percentage relative abundance in at least one of the enrichments (1st, 2nd, or 3rd month) of the same type (DIC, **A**; IBU, **B**; or CBZ, **C**) compared to the percentage relative abundance in the initial biofilm bacterial community

We also identified bacterial genera with ≥ 0.5% and an at least 100-fold increase in percentage relative abundance in at least one of the enrichment cultures compared to their initial values in the starting biofilm bacterial community. For DIC enrichments, by the end of the third month, the highest increase in percentage relative abundance was detected for *Ferrovibrio*, *Hydrocarboniphaga*, *Zavarzinia*, and *Sphingopyxis* showing a 1005-, 957-, 811-, and 628-fold increase, respectively (Fig. 3A; Online Resource 2). In the IBU-amended enrichments, the highest percentage relative abundance increase was recorded for the genera *Starkeya* (a 1002-fold increase), *Methylibium* (a 314-fold increase), and a so far uncultivated bacterial taxa affiliating with the metagenome-assembled genome *bacterium SCN 62–11* (a 350-fold increase; Fig. 3B; Online Resource 2). Noteworthy, the highest percentage relative abundance of the genus *Pimelobacter* in the IBU enrichments was only 0.18%, yet it showed a 1000-fold increase by the end of the IBU enrichment. Moreover, already in the first 2 months, the genus showed a ~ 2000-fold increase compared to initial percentage relative abundance values. In the CBZ-amended enrichments, one of the most conspicuous percentage relative abundance increases were found in the case of the genera *Pseudonocardia*, *Sphingopyxis*, and *Rhodococcus*, showing a 505-, 435-, and 220-fold increase, respectively (Fig. 3C; Online Resource 2).

Similar to the IBU enrichment, *Pimelobacter* showed the highest, a close to 2700-fold, increase by the end of the 3rd month, although the percentage relative abundance of the genus was at most 0.32% in all the CBZ enrichments. Already after two months of CBZ enrichment, the genus showed a more than a 4000-fold increase in percentage relative abundance, which halved by the end of the third month.

Surprisingly, bacterial genera that were the most dominant (Fig. 2, Genus) such as *Pseudomonas*, *Methyloversatilis*, and *Azospirillum* showed only an 11- and 60-fold increase in percentage relative abundance by the end of the enrichments.

### Reconstruction of metagenome-assembled bacterial genomes

From three additional metagenomes of enrichment cultures after three months with higher sequencing depth, the genomes of most bacteria that showed the highest percentage relative abundance increase during the enrichments were reconstructed such as *Ferrovibrio* sp., *Zavarzinia* sp., *Prosthecobacter* sp., *Rhodanobacter thiooxydans*, *Pseudonocardia* sp., *Sphingopyxis* sp., *Rhodococcus* sp., and *Achromobacter* sp. (Fig. 3; Table 1). Metabolic analyses of these bacteria indicated that they encode for genes involved in the biodegradation of xenobiotics such as benzoate/aminobenzoate, simple aromatic hydrocarbons (e.g., toluene, xylene, ethyl-benzene) and PAHs (e.g., naphthalene), halogenated compounds (e.g., chlorinated alkanes and alkanes, chlorocyclohexane, etc.), steroids, and even drugs which biodegradation is governed by the cytochrome P450 enzyme system (Online Resource 3). No bacterial genomes affiliating with the genera *Hydrocarboniphaga*, *Starkeya*, and *Pimelobacter* were reconstructed.

**Table 1:** Completeness and accession numbers of reconstructed bacterial genomes of those bacterial genera that showed the highest percent relative abundance increase during the enrichments (8 bins)

	checkM* completeness (%)	checkM* contamination (%)	GC (%)	Size (Mbps)	Enrichment type	Fold percent relative abundance increase	NCBI accession number
*Ferrovibrio* sp. bin D_3	94.4	1.6	63.8	4.9	DIC	1005	JAOZVR000000000
*Zavarzinia* sp. bin D_3	82.3	0.4	67.0	3.5	DIC	811	JAOZVS000000000
*Prosthecobacter* sp. bin D_3	86.8	2.4	53.7	5.5	DIC	226	JAOZVT000000000
*Rhodanobacter thiooxydans* bin D_3	89.5	9.8	67.2	4.1	DIC	130	JAOZVU000000000
*Pseudonocardia* sp. bin C_3	93.1	1.6	73.3	5.3	CBZ	505	JAOZVV000000000
*Sphingopyxis* sp. bin C_3	99.7	1.3	66.4	4	CBZ	435	JAOZVW000000000
*Rhodococcus* sp. bin C_3	75.3	0.8	62.3	5.8	CBZ	220	JAOZVY000000000
*Achromobacter* sp. bin C_3	96.0	3.4	67.8	6.5	CBZ	135	JAOZVX000000000

^*^Parks et al. (2015)

### Bacterial diversity of enrichment cultures based on cultivation-dependent approach

In total, 31 bacterial isolates were obtained, ten from the DIC, nine from the IBU, and twelve from the CBZ enrichment (Table 2). At the class level, the isolates belonged to the class of Gammaproteobacteria (14 isolates), Alphaproteobacteria (7), Betaproteobacteria (7), Actinobacteria and Bacilli (2). The majority of isolates (13) affiliated with the genus *Pseudomonas* (DIC, 4; IBU, 5; and CBZ, 4 isolates). *Pseudomonads* were followed by representatives of the genera *Rhizobium* (DIC, 3, and IBU, 2 isolates), *Methyloversatilis* (1 isolate from each enrichment), *Nocardioides* (only CBZ enrichment, 2 isolates), *Variovorax* (only CBZ enrichment, 2 isolates) and *Bacillus* (IBU, 1, and CBZ, 1 isolates). One isolate each was obtained from the genera *Stenotrophomonas* (DIC enrichment), *Ancylobacter* (IBU), *Brevundimonas* (CBZ) and *Rhodococcus* (CBZ). Out of all isolates, 26 belonged to the group of Gram (-) and five to Gram ( +) bacteria.

**Table 2:** Taxonomic affiliation of the isolated strains obtained from the 1-, 2-, and 3-month-old DIC, IBU, and CBZ amended enrichments

Name of isolates	Nearest cultured neighbour based on *16S rRNA* gene	Phylogenetic affiliation	bp	*16S rRNA* gene sequence homology (%)	*16S rRNA* gene sequence accession numbers	Enrichment
DIC_1	*Pseudomonas stutzeri* ATCC 17588^ T^	Gammaproteobacteria	1434	99.23	OM818629	DIC, 1st month
DIC_5	*Stenotrophomonas humi* DSM 18929^ T^	Gammaproteobacteria	1444	99.58	OM818630	
DIC_8	*Pseudomonas kribbensis* 46-2^ T^	Gammaproteobacteria	1443	99.51	OM818631	
DIC_11	*Bacillus mobilis* 0711P9-1^ T^	Bacilli	1460	99.72	OM818632	DIC, 2nd month
DIC_12	*Methyloversatilis discipulorum* FAM1^T^	Betaproteobacteria	1436	100.0	OM818633	
DIC_14	*Rhizobium daejeonense* KCTC 12121^ T^	Alphaproteobacteria	1396	99.35	OM818634	
DIC_15	*Rhizobium daejeonense* KCTC 12121^ T^	Alphaproteobacteria	1387	99.13	OM818635	
DIC_16/A	*Rhizobium daejeonense* KCTC 12121^ T^	Alphaproteobacteria	1385	99.13	OM818636	DIC, 3rd month
DIC_17	*Pseudomonas kribbensis* 46-2^ T^	Gammaproteobacteria	1434	99.44	OM818637	
DIC_18	*Pseudomonas stutzeri* ATCC 17588^ T^	Gammaproteobacteria	1436	99.02	OM818638	
IBU_1	*Pseudomonas stutzeri* ATCC 17588^ T^	Betaproteobacteria	1435	99.23	OM818639	IBU, 1st month
IBU_2	*Ancylobacter defluvii* SK15^T^	Actinobacteria	1381	**97.88**	OM818640	
IBU_4	*Methyloversatilis discipulorum* FAM1^T^	Betaproteobacteria	1427	99.86	OM818641	
IBU_9	*Pseudomonas kribbensis* 46-2^ T^	Gammaproteobacteria	1419	99.42	OM892062	
IBU_12	*Pseudomonas veronii* DSM11331^T^	Gammaproteobacteria	1437	99.86	OM818642	
IBU_13	*Pseudomonas stutzeri* ATCC 17588^ T^	Gammaproteobacteria	1449	99.93	OM818643	IBU, 2nd month
IBU_14	*Rhizobium daejeonense* KCTC 12121^ T^	Alphaproteobacteria	1385	99.35	OM818644	
IBU_17	*Pseudomonas stutzeri* ATCC 17588^ T^	Gammaproteobacteria	1440	99.23	OM818645	IBU, 3rd month
IBU_18	*Rhizobium daejeonense* KCTC 12121^ T^	Alphaproteobacteria	1395	99.05	OM818646	
CBZ_1*	*Nocardioides carbamazepini* CBZ_1^T^	Actinobacteria	1411	100	OM818617	CBZ, 1st month
CBZ_2	*Nocardioides carbamazepini* CBZ_1^T^	Actinobacteria	1415	100	OM818618	
CBZ_3	*Brevundimonas mongoliensis* R-10-10^ T^	Alphaproteobacteri	1368	99.11	OM818619	
CBZ_4	*Pseudomonas kribbensis* 46-2^ T^	Gammaproteobacteria	1376	99.64	OM818620	
CBZ_6	*Rhodococcus qingshengii* JCM 15477^ T^	Actinobacteria	1399	100.0	OM818621	
CBZ_7	*Pseudomonas moorei* RW10^T^	Gammaproteobacteria	1387	99.19	OM818622	
CBZ_8	*Pseudomonas veronii* DSM11331^T^	Gammaproteobacteria	1426	99.58	OM818623	
CBZ_9	*Variovorax paradoxus* NBRC 15149^ T^	Betaproteobacteria	1442	99.23	OM818624	
CBZ_10	*Variovorax paradoxus* NBRC 15149^ T^	Betaproteobacteria	1430	99.23	OM818625	CBZ, 2nd month
CBZ_11	*Methyloversatilis discipulorum* FAM1^T^	Betaproteobacteria	1301	100.0	OM818626	
CBZ_13	*Pseudomonas stutzeri* ATCC 17588^ T^	Gammaproteobacteria	1425	99.13	OM818627	CBZ, 3rd month
CBZ_16	*Bacillus cereus* ATCC 14579^ T^	Bacilli	1449	99.79	OM818628	

^*^We recently described isolate CBZ_1^T^ as a new phyletic lineage within the genus *Nocardioides* to which the name *Nocardioides carbamazepini* was proposed (Benedek et al. 2022)

Bacterial isolations resulted in two new bacterial species from the IBU and CBZ enrichments belonging to the genera *Ancylobacter* and *Nocardioides*, respectively. *16S rRNA* genes of isolates *Nocardioides* sp. nov. CBZ_1 and CBZ_2, and *Ancylobacter* sp. nov. IBU_2 showed below 98% sequence similarity with *16S rRNA* genes of the nearest cultured neighbors (Table 2). The *16S rRNA* gene sequence similarity cutoff value for the demarcation of new bacterial species is 98.7% (Stackebrandt and Ebers 2006) or 98.65% (Kim et al. 2014). Isolate CBZ_1^T^ has recently been described by the authors as a new phyletic lineage within the genus *Nocardioides* to which the name *Nocardioides carbamazepini* was given (Benedek et al. 2022).

### Determination of pharmaceutical biodegradation ability of isolates

According to the screening assays, the added pharmaceutical compounds exerted different effects on the tested organisms. In some cases, the presence of the added carbon source (DIC, IBU, or CBZ) inhibited the bacterial growth, while in other cases, the bacterial growth was remarkably increased by the presence of the compounds. There were a few examples when the added carbon sources apparently had no significant effect on the activity of the tested organisms. These isolates tolerated the presence of pharmaceutical compounds, rather than utilized them as carbon sources. Out of the 31 isolated strains, 16 isolates proved to be tolerant to the presence of pharmaceuticals, the growth of eight isolates was inhibited by the compounds and seven isolates were marked as potential degraders (Online Resource 4).

Among the isolates from the DIC enrichments, *S. humi* DIC_5 and *R. daejeonense* strain DIC_15 were selected as potential DIC degraders. *R. daejeonense* strains IBU_14 and IBU_18, isolated from the 2nd and 3rd month IBU enrichments, were marked as potential IBU degraders. Among the CBZ enrichment derived isolates, 3 strains were labelled as potential pharmaceutical degraders: *N. carbamazepini* CBZ_1, *B. bullata* CBZ_3 and *V. paradoxus* CBZ_10 (Online Resource 4).

HPLC measurements unequivocally confirmed the pharmaceutical biodegradation capacity of only *S. humi* DIC_5 and *R. daejeonense* IBU_18 isolates. After four weeks of incubation, strain DIC_5 almost completely degraded DIC (91 ± 0.034% concentration reduction) in the presence of glucose (3 g l^−1^); no higher biodegradation rate could be reached under other testing conditions. After 4 weeks of incubation, isolate IBU_18 showed the highest 90.7 ± 0.098% IBU biodegradation rate in R2A broth; in the yeast-extract amended (0.03 g l^−1^) test solution the IBU biodegradation rate was only 53 ± 0.58%. For other potential pharmaceutical degraders, only a 10–20% pharmaceutical biodegradation capacity could be determined using HPLC.

## Discussion

In this study, the identification, isolation and testing of DIC, IBU, and CBZ degrading bacteria from a bacterial biofilm was conducted for future biodegradation purposes. Preliminary work had identified the inoculum biofilm bacterial community as a source of versatile xenobiotic degrading bacteria (BTEX and PAH degraders) with biotechnological relevance (Benedek et al. 2016; 2018 and 2020).

A bacterial strain collection with 31 isolates was obtained (Table 2). Promising bacterial taxa which showed high relative abundance or a high relative abundance increase during the enrichments were cultivated, or their genomes were assembled and studied using metagenome binning and metabolic analyses, respectively.

At the genus level, the enrichment cultures were mostly dominated by two genera *Methyloversatilis* and *Pseudomonas*; however, the increase in percentage relative abundance of these two genera throughout the enrichment period was negligible.

Up to this point, there was no information regarding the capacity of pharmaceutical biodegradation by organisms of the genus *Methyloversatilis*. The genus was described in 2006 with the type species *M. universalis* FAM5^T^ as a facultative methylotroph Betaproteobacterium (Kalyuzhnaya et al. 2006). The obtained isolates affiliating with the genus *Methyloversatilis* did not show pharmaceutical biodegradation capacity in this study. As for *Pseudomonas*, a few studies have reported the pharmaceutical biodegradation capacity of the genus including DIC and CBZ (Jiang et al. 2014; Li et al. 2015a, b; Hemidouche et al. 2018; Hu et al. 2013; Li et al. 2013; Lin et al. 2015; Daou et al. 2020). This is not surprising because members of the genus *Pseudomonas* have been known to be metabolically versatile and to be involved in the degradation of different xenobiotics (Arenghi et al. 2001; Hendrickx et al. 2005; Wang et al. 2008; Ajithkumar et al. 2003; Nam et al. 2003; Hong et al. 2004; Onaca et al. 2007). Surprisingly, in our study, the obtained *Pseudomonas* isolates either proved to be pharmaceutical tolerant or their growth was inhibited by the presence of the tested pharmaceuticals (Online Resource 4). The explanation for this contradictory finding could be that during the tests the applied pharmaceutical concentrations were high or *Pseudomonas* spp. require co-metabolic substrates for the biodegradation of these compounds.

In DIC enrichments, other notable genera were identified and included *Azospirillum*, *Rhodanobacter*, *Ferrovibrio*, *Hydrocarboniphaga*, *Zavarzinia*, *Sphingopyxis*, and *Prosthecobacter* showing either a high relative abundance or a significant increase in relative abundance during the enrichments. With regard to *Azospirillum*, little evidence exists in the literature regarding the pharmaceutical biodegradation capacity of the genus. In the study of Navrozidou et al. (2019b), investigating the microbiota of an immobilized cell biofilter treating DIC-based wastewater, the genus *Azospirillum* was named amongst major bacterial taxa, although its percentage relative abundance within the community was only 0.5%. The possible involvement of the genus *Rhodanobacter* in pharmaceutical biodegradation seemed to be supported by previous studies. Navrozidou et al. (2019a and 2019b) identified *Rhodanobacter* as dominant part of the IBU and DIC degrading microbiota in an immobilized cell bioreactor. Organisms related to *Ferrovibrio* showed the highest relative abundance increase in the DIC amended enrichments, i.e., by 1000-fold (Fig. 3A). The genus was described in 2013 with the type species *F. denitrificans* Sp-1^ T^ as a neutrophilic, iron oxidizing vibrio, isolated from the redox zone of a low-salinity spring in Krasnodar krai (Russia), at the FeS-Fe(OH)_3_ interface deposited at the sediment surface (Sorokina et al. 2012). Apart from *F. denitrificans*, the genus contains three additional described species *F. soli*, *F. terrae*, and *F. xuzhouensis. F. xuzhouensis* LM-6^ T^ was isolated from a cyhalothrin contaminated wastewater and is capable of cyhalothrin-degradation (Song et al. 2015). To date, no studies have been published reporting the capacity for biodegradation of DIC or any other pharmaceutical compound for this genus, although cyhalothrin and DIC have similar molecular structures. To the best of our knowledge, there is also no information regarding the pharmaceutical biodegradation capacity of isolates affiliating with the genera *Hydrocarboniphaga* and *Zavarzinia.* By contrast, there is evidence that members of the genus *Sphingopyxis* may be capable of pharmaceutical biodegradation. *Sphingopyxis granuli* strain RW412 isolated from the sediment of the river Elbe completely removed IBU (2 mM) from a biopurification system. *S. granuli* RW412 harbors in its genome the *ipf* (IBU biodegradation gene cluster) genes that encode the first steps of IBU mineralization (Aguilar-Romero et al. 2021). Regarding the genus *Prosthecobater*, previous studies have also reported the high prevalence of *Prosthecobacter* in microbial communities treated with pharmaceuticals and personal care products such as IBU, naproxen, prednisolone, norfloxacin, CBZ, and sulfamethoxazole (Zhao et al. 2015; Tiwari et al. 2019; Rutere et al. 2020). Although they could not be isolated, genomes of *Rhodanobacter*, *Ferrovibrio*, *Zavarzinia*, *Prosthecobacter*, and *Sphingopyxis* species could be reconstructed from metagenomes. The metabolic analyses of the genomes showed that these bacteria possess genes responsible for xenobiotics biodegradation and pharmaceutical biodegradation; genes involved in benzoate, aminobenzoate, chloroalkane/chloroalkene, chlorocylohexane/chlorobenzene, dioxin, and nitrotoluene biodegradation were identified (Thelusmond et al. 2019; Balcom et al. 2016; Table 1; Online Resource 3). It is noteworthy that the genome affiliating with the genus *Sphingopyxis* was reconstructed only from the CBZ supplemented enrichment.

In the IBU-amended enrichment cultures, representatives of the genera *Starkey*a, *Methylibium*, *Rhodococcus*, and *bacterium SCN 62–11* deserve further discussion. So far, only one study has reported the involvement of the genus *Starkeya* in pharmaceutical biodegradation. Bessa et al. (2017) isolated a CBZ degrader *Starkeya* sp. strain C11 from the activated sludge of a municipal WWTP. The genus *Methylibium,* a known hydrocarbon degrader, was also most probably involved in the biodegradation of pharmaceuticals in the study of Tiwari et al. (2019); *Methylibium* increased in a submerged membrane bioreactor treating synthetic hospital wastewater containing IBU, CBZ, estradiol, and venlafaxine. While reported cases of *Starkeya* and *Methylibium* degrading pharmaceuticals are very limited, lots of studies have reported pharmaceutical biodegradation capacity of the genus *Rhodococcus*. For instance, *R. rhodochrous* ATCC 13,808 degraded CBZ, sulfamethoxazole, and sulfamethizole (Gauthier et al. 2010); *R. rhodochrous* IEGM 608 degraded drotaverine (Ivshina et al. 2012), *R. ruber* strain IEGM 346 completely degraded DIC (50 µg l^−1^) within over 6 days (Ivshina et al. 2019); *R. zopfii* Y50158 degraded 17β-estradiol (Yoshimoto et al. 2004); both, *R. erythropolis* ATCC 4277 and *R. zopfii* ATCC 51,349 were capable of degrading 17α-ethinylestradiol (O’Grady et al. 2009; Menashe et al. 2020). The metagenomic bin *bacterium SCN 62–11* was detected for the first time in a thiocyanate (SCN–) degrading microbial community developed in a laboratory-scale bioreactor (Kantor et al. 2015). No information has been published regarding the involvement of this bacterium in the biodegradation of pharmaceuticals.

In the CBZ enrichments, the highest increase in relative abundance were detected in the case of genera *Pseudonocardia*, *Sphingopyxis*, *Rhodococcus*, and *Achromobacter* (Fig. 3). There is almost no information regarding pharmaceutical biodegradation capacity of the genus *Pseudonocardia*. Thelusmond et al. (2018) reported that *Pseudonocardia* were amongst the top 25 bacterial taxa in triclocarban (antibiotic) amended soils. It is noteworthy that xenobiotic biodegradation capacity of the genus is well documented (Vainberg et al. 2006; Chen et al. 2018; Masuda et al. 2012; Yamamoto et al. 2018; Kim et al. 2010). The role of *Sphingopyxis* and *Rhodococcus* in the biodegradation of pharmaceuticals have been discussed above. As for the *Achromobacter*, to date, no studies have been reported discussing DIC, IBU or CBZ biodegradation potential of the genus. However, according to several studies, *Achromobacter* affiliating isolates are capable of antibiotic biodegradations such as sulfamethoxazole and ceftriaxone (Reis et al. 2014; Liang et al. 2021; Anan et al. 2017). The reconstructed bacterial genomes affiliating with the genera *Pseudonocardia*, S*phingopyxis*, *Rhodococcus*, and *Achromobacter* encode genes involved in xenobiotics biodegradation including drugs and steroids.

Other bacterial genera which deserve discussion in this study are *Variovorax*, *Rhizobium*, *Nocardioides*, as well as *Pimelobacter*. Although *Variovorax*, *Rhizobium*, and *Nocardioides* did not increase notable in relative abundance during the enrichments, they were most probably key bacterial community members in DIC, IBU, or CBZ enrichments (Fig. 2). Although members of the genus *Variovorax* are metabolically versatile (Satola et al. 2013), there is only one study reporting the pharmaceutical biodegradation capacity of the genus. Murdoch and Hay (2015) isolated a *Variovorax* sp. strain Ibu-1 from activated sludge with the ability to use IBU as sole source of carbon and energy. The involvement of the genus *Rhizobium* in pharmaceuticals biodegradation has been proven by Bessa et al. (2017) who isolated the CBZ degrading *Rhizobium* sp. strain C12. Vasiliadou et al. (2018) showed the dominance of the genus *Rhizobium* (21%) in a mixed microbial community of activated sludge enriched on caffeine, sulfamethoxazole and CBZ. Although it has been proven that many *Nocardioides* strains play an important role in the biodegradation of a variety of pollutants, including alkanes, pyridine, phenols, phenanthrene, herbicides (for a detailed review about the genus *Nocardioides* see Yoon and Park 2006), there is little information about pharmaceutical biodegradation capacity of the genus *Nocardioides*. In the study of Rutere et al. (2020), *Nocardioides* were amongst major bacterial taxa enriched from oxic hyporheic zone sediment bacterial communities in response to IBU. In the study of Posselt et al. (2020), bacterial taxa in the sediment collected from the river Erpe that were associated with the biodegradation of 31 different compounds (mainly pharmaceuticals) in river-simulating flumes, included *Nocardiodies*. Chen et al. (2019) reported *Nocardioides* as potential sulfadiazine (antibiotic) degraders. We recently described strain CBZ_1^T^ as *Nocardioides carbamazepini* as a new phyletic lineage within the genus *Nocardioides* capable of IBU biodegradation (Benedek et al. 2022).

Among all taxa, the genus *Pimelobacter* showed the highest relative abundance increase in both IBU and CBZ amended enrichments. The genus *Pimelobacter* from the family of *Nocardioidaceae* was described by Suzuki and Komagata (1983) with the type species *Pimelobacter simplex* CNF 035^ T^. The results of metagenome sequencing and phylogenetic analyses of IBU and CBZ enrichments revealed that at the species level all *Pimelobacter* related sequence reads affiliated with *P. simplex*. It is worth mentioning that *P. simplex* was transferred later to the genus *Nocardioides* (O'Donnell et al. 1982; Collins et al. 1989). Therefore, it can be assumed that in the IBU and CBZ amended enrichments the bacterial taxa that showed the highest increase in relative abundance affiliated with the genus *Nocardioides* and members of this genus are most probably capable of IBU and CBZ degradation.

## Concluding remarks

We showed that the investigated groundwater biofilm harbored bacteria capable of pharmaceutical biodegradation. The autochthonous groundwater bacterial community possesses the inherent ability to cope with the most common pharmaceutical residues in aquatic ecosystems. We have shown that most probably members of the genera *Nocardioides* and *Ferrovibrio* are involved in the biodegradation of IBU and DIC, respectively. Using metagenome binning, the genomes of those bacteria that showed the highest relative abundance increase during the enrichments such as *Ferrovibrio*, *Zavarzinia*, *Prosthecobacter*, *Rhodanobacter*, *Pseudonocardia*, *Sphingopyxis*, *Rhodococcus*, and *Achromobacter* could be assembled and were most probably involved in the biodegradation of the studied PhACs. At least two bacterial isolates *Stenotrophomonas humi* strain DIC_5 and *Rhizobium daejeonense* strain IBU_18 could be isolated that showed a remarkable DIC and IBU biodegradation ability, respectively. After 4 weeks of incubation, strain DIC_5 almost completely eliminated DIC (91 ± 0.034% concentration reduction) in the presence of glucose (3 g l^−1^); isolate IBU_18 degraded 90.77 ± 0.098% of the initial IBU concentration in R2A broth.

It can be concluded that metagenomic binning conducted in this study further uncovered the microbial dark matter of PhACs degrading bacteria. Last but not least, this research provides a platform for other scientists by showing results of a reference nature.

The findings of this study project new research directions. In the future, it would be worth investigating (i) the biodegradation capacity of the isolated strains at lower PhACs concentrations and with the inclusion of other groups of PhACs; (ii) the testing of DIC and IBU biodegradation capacity of strains DIC_5 and IBU_18 under close to environmental conditions using natural water samples or wastewater effluents in the presence of the autochthonous microbial community; (iii) if complete mineralization or only biotransformation occurs during the biodegradation of the tested pharmaceuticals by the isolated strains; (iv) the possibility to engineer from the isolated strain a bacterial consortium capable of simultaneous biodegradation of DIC, IBU, and CBZ under environmental conditions; (v) the possibility of isolation and testing of those bacterial genera that showed the highest relative abundance increase during the enrichments and whose genome has been already assembled using bioinformatic tools (*Ferrovibrio*, *Hydrocarboniphaga*, *Zavarzinia*, *Starkeya*, *Pseudonocardia*, and *Sphingopyxis*).

## Supplementary Information

Below is the link to the electronic supplementary material.

Supplementary file1 (DOCX 16 KB)

Supplementary file2 (DOCX 16 KB)

Supplementary file3 (XLSX 63 KB)

Supplementary file4 (DOCX 21 KB)

## Data Availability

All data generated or analyzed during this study are included in this published article or can be found in the National Center for Biotechnology Information (NCBI) on the basis of the provided accession numbers. **Ethical approval.** Ethical approval and informed consent were not required for this study.
