# Supplementary material for: Selective enrichment, identification, and isolation of diclofenac, ibuprofen, and carbamazepine degrading bacteria from a groundwater biofilm
Source: Environ Sci Pollut Res Int. 2023 Jan 24;30(15):44518–35. doi: 10.1007/s11356-022-24975-6 (PMC10076411; doi:10.1007/s11356-022-24975-6)
Supplement: Supplementary file 1 — Supplementary file1 (DOCX 16 KB) [file 11356_2022_24975_MOESM1_ESM.docx]

**Selective enrichment, identification and isolation of diclofenac, ibuprofen and carbamazepine degrading bacteria from a groundwater biofilm**

Márton Pápai^a¥^, Tibor BENEDEK^a*¥^, András Táncsics^a^, Till L.V. Bornemann^b^, Julia Plewka^b^, Alexander J. Probst^b^, Daood Hussein^c^, Gergely Maróti^d,e^, Ofir MENASHE^f,g^ Balázs Kriszt^h^,

^a^Hungarian University of Agriculture and Life Sciences, Institute of Aquaculture and Environmental Safety, Department of Molecular Ecology, Gödöllő, H-2100, Páter K. u. 1, Hungary;

*^b^Group for Aquatic Microbial Ecology, Environmental Microbiology and Biotechnology, Faculty of Chemistry, University of Duisburg-Essen, Essen, Universitäts str. 5, 45141 Essen, Germany*

*^c^Institute of Horticultural Sciences, Laboratories of Food Analysis, Hungarian University of Agriculture and Life Sciences, Gödöllő, Hungary*

*^d^Institute of Plant Biology, Biological Research Center of the Hungarian Academy of Sciences, Temesvári krt. 62., Szeged, Hungary*

*^e^Seqomics Biotechnology Ltd., Mórahalom, Hungary*

*^f^Water Industry Engineering Department, The Engineering Faculty, , Kinneret Academic College on the Sea of Galilee,* *D.N. Emek Ha'Yarden 15132, Israel*

*^g^BioCastle Water Technologies Ltd.,* *Tzemah, Israel*

*^h^Hungarian University of Agriculture and Life Sciences, Institute of Aquaculture and Environmental Safety, Department of Environmental Safety, Gödöllő, H-2100, Páter K. u. 1, Hungary;*

^¥^ Both authors contributed equally to this work

Journal: Environmental Science and Pollution Research

*E-mail address of the corresponding author: [benedektibor001@gmail.com](mailto:benedektibor001@gmail.com)

The number of *Bacteria* related metagenome sequence reads used to determine phylogenetic diversity of the samples

| **Sample designation** | **Sample Type** | | **Nr. of bps** | **Sequence Read Accession (SRA) numbers** |
| --- | --- | --- | --- | --- |
| **BF** | | Initial Biofilm | 1 266 554 | SRR17027096 |
| **D_1** | | DIC 1^st^ month | 2 316 533 | SRR17049155 |
| **I_1** | | IBU 1^st^ month | 1 940 477 | SRR17049152 |
| **C_1** | | CBZ 1^st^ month | 1 762 731 | SRR17049149 |
| **D_2** | | DIC 2^nd^ month | 2 308 433 | SRR17049154 |
| **I_2** | | IBU 2^nd^ month | 2 121 575 | SRR17049151 |
| **C_2** | | CBZ 2^nd^ month | 2 293 835 | SRR17049148 |
| **D_3** | | DIC 3^rd^ month | 2 356 073 | SRR17049153 |
| **I_3** | | IBU 3^rd^ month | 2 091 048 | SRR17049150 |
| **C_3** | | CBZ 3^rd^ month | 2 183 933 | SRR17049147 |
