# Supplementary material for: Selective enrichment, identification, and isolation of diclofenac, ibuprofen, and carbamazepine degrading bacteria from a groundwater biofilm
Source: Environ Sci Pollut Res Int. 2023 Jan 24;30(15):44518–35. doi: 10.1007/s11356-022-24975-6 (PMC10076411; doi:10.1007/s11356-022-24975-6)
Supplement: Supplementary file 2 — Supplementary file2 (DOCX 16 KB) [file 11356_2022_24975_MOESM2_ESM.docx]

**Selective enrichment, identification and isolation of diclofenac, ibuprofen and carbamazepine degrading bacteria from a groundwater biofilm**

Márton Pápai^a¥^, Tibor BENEDEK^a*¥^, András Táncsics^a^, Till L.V. Bornemann^b^, Julia Plewka^b^, Alexander J. Probst^b^, Daood Hussein^c^, Gergely Maróti^d,e^, Ofir MENASHE^f,g^ Balázs Kriszt^h^,

^a^Hungarian University of Agriculture and Life Sciences, Institute of Aquaculture and Environmental Safety, Department of Molecular Ecology, Gödöllő, H-2100, Páter K. u. 1, Hungary;

*^b^Group for Aquatic Microbial Ecology, Environmental Microbiology and Biotechnology, Faculty of Chemistry, University of Duisburg-Essen, Essen, Universitäts str. 5, 45141 Essen, Germany*

*^c^Institute of Horticultural Sciences, Laboratories of Food Analysis, Hungarian University of Agriculture and Life Sciences, Gödöllő, Hungary*

*^d^Institute of Plant Biology, Biological Research Center of the Hungarian Academy of Sciences, Temesvári krt. 62., Szeged, Hungary*

*^e^Seqomics Biotechnology Ltd., Mórahalom, Hungary*

*^f^Water Industry Engineering Department, The Engineering Faculty, , Kinneret Academic College on the Sea of Galilee,* *D.N. Emek Ha'Yarden 15132, Israel*

*^g^BioCastle Water Technologies Ltd.,* *Tzemah, Israel*

*^h^Hungarian University of Agriculture and Life Sciences, Institute of Aquaculture and Environmental Safety, Department of Environmental Safety, Gödöllő, H-2100, Páter K. u. 1, Hungary;*

^¥^ Both authors contributed equally to this work

Journal: Environmental Science and Pollution Research

*E-mail address of the corresponding author: [benedektibor001@gmail.com](mailto:benedektibor001@gmail.com)

Bacterial genera with potential pharmaceutical biodegradation capacity that showed the highest increase in percent relative abundance by the end of the 3^rd^ month of enrichment compared to the percent relative abundance of the genus within the initial biofilm bacterial community.

| **Genera** | **Increase in percent relative abundance by the end of the 3^rd^ month of enrichment**  **(-fold increase)** | | |
| --- | --- | --- | --- |
|  | **DIC** | **IBU** | **CBZ** |
| *Achromobacter* |  |  | **135** |
| *Ferrovibrio* | **1005** |  |  |
| *Hydrocarboniphaga* | **957** |  |  |
| *Methylibium* |  | **314** |  |
| *Pimelobacter* |  | **997** | **2692** |
| *Prosthecobacter* | **226** |  |  |
| *Pseudonocardia* |  |  | **505** |
| *Sphingopyxis* | **628** |  | **435** |
| *Zavarzinia* | **811** |  |  |
| *Afipia* | **184** |  |  |
| *Rhodanobacter* | **130** |  |  |
| *Starkeya* |  | **1002** |  |
| *Rhodococcus* |  | **93** | **220** |
| *bacterium SCN 62-11* |  | **350** |  |
