# Supplementary material for: Selective enrichment, identification, and isolation of diclofenac, ibuprofen, and carbamazepine degrading bacteria from a groundwater biofilm
Source: Environ Sci Pollut Res Int. 2023 Jan 24;30(15):44518–35. doi: 10.1007/s11356-022-24975-6 (PMC10076411; doi:10.1007/s11356-022-24975-6)
Supplement: Supplementary file 4 — Supplementary file4 (DOCX 21 KB) [file 11356_2022_24975_MOESM4_ESM.docx]

**Selective enrichment, identification and isolation of diclofenac, ibuprofen and carbamazepine degrading bacteria from a groundwater biofilm**

Márton Pápai^a¥^, Tibor BENEDEK^a*¥^, András Táncsics^a^, Till L.V. Bornemann^b^, Julia Plewka^b^, Alexander J. Probst^b^, Daood Hussein^c^, Gergely Maróti^d,e^, Ofir MENASHE^f,g^ Balázs Kriszt^h^,

^a^Hungarian University of Agriculture and Life Sciences, Institute of Aquaculture and Environmental Safety, Department of Molecular Ecology, Gödöllő, H-2100, Páter K. u. 1, Hungary;

*^b^Group for Aquatic Microbial Ecology, Environmental Microbiology and Biotechnology, Faculty of Chemistry, University of Duisburg-Essen, Essen, Universitäts str. 5, 45141 Essen, Germany*

*^c^Institute of Horticultural Sciences, Laboratories of Food Analysis, Hungarian University of Agriculture and Life Sciences, Gödöllő, Hungary*

*^d^Institute of Plant Biology, Biological Research Center of the Hungarian Academy of Sciences, Temesvári krt. 62., Szeged, Hungary*

*^e^Seqomics Biotechnology Ltd., Mórahalom, Hungary*

*^f^Water Industry Engineering Department, The Engineering Faculty, , Kinneret Academic College on the Sea of Galilee,* *D.N. Emek Ha'Yarden 15132, Israel*

*^g^BioCastle Water Technologies Ltd.,* *Tzemah, Israel*

*^h^Hungarian University of Agriculture and Life Sciences, Institute of Aquaculture and Environmental Safety, Department of Environmental Safety, Gödöllő, H-2100, Páter K. u. 1, Hungary;*

^¥^ Both authors contributed equally to this work

Journal: Environmental Science and Pollution Research

*E-mail address of the corresponding author: [benedektibor001@gmail.com](mailto:benedektibor001@gmail.com)

Results of the resazurin assay, selection of potential pharmaceutical degraders for HPLC analyses

| **Tested Isolates** | **Absorbance of samples measured at 610 nm ± STD**  **(means of three replicates ± STD ^α^)** | | | |
| --- | --- | --- | --- | --- |
|  | **Abiotic Control ^β^** | **Blank ^γ^** | **Sample ^δ^** | **The result of pre screenings** |
| **Diclofenac Enrichment** |  |  |  |  |
| *P. stutzeri* DIC-1 |  | 0.100 ± 0.007 | 0.144 ± 0.004 | Growth inhibition ^ε^ |
| ***S. humi* DIC-5** |  | **0.051 ± 0.016** | **0.012 ± 0.022** | **Potential degrader ^θ^** |
| *P. kribbensis* DIC-8 |  | 0.146 ± 0.002 | 0.135 ± 0.003 | Pharmaceutical tolerant ^λ^ |
| *B. mobilis* DIC-11 |  | 0.144 ± 0.004 | 0.147 ± 0.004 | Pharmaceutical tolerant |
| *M. discipulorum* DIC-12 | 0.146 ± 0.01 | 0.073 ± 0.010 | 0.084 ± 0.012 | Pharmaceutical tolerant |
| *R. daejeonense* DIC-14 |  | 0.018 ± 0.001 | 0.087 ± 0.022 | Growth inhibition |
| ***R. daejeonense* DIC-15** |  | **0.074 ± 0.001** | **0.035 ± 0.023** | **Potential degrader** |
| *R. daejeonense* DIC-16/A |  | 0.013 ± 0.004 | 0.018 ± 0.009 | Pharmaceutical tolerant |
| *P. kribbensis* DIC-17 |  | 0.014 ± 0.001 | 0.119 ± 0.004 | Growth inhibition |
| *P. stutzeri* DIC-18 |  | 0.045 ± 0.008 | 0.119 ± 0.010 | Growth inhibition |
| **Ibuprofen Enrichment** |  |  |  |  |
| *P. stutzeri* IBU-1 | 0.146 ± 0.01 | 0.140 ± 0.0015 | 0.138 ± 0.006 | Pharmaceutical tolerant |
| *Ancylobacter* sp. nov. IBU-2 |  | 0.145 ± 0.0035 | 0.146 ± 0.006 | Pharmaceutical tolerant |
| *M. discipulorum* IBU-4 |  | 0.130 ± 0.002 | 0.134 ± 0.002 | Pharmaceutical tolerant |
| *P. kribbensis* IBU-9 |  | 0.142 ± 0.000 | 0.146 ± 0.002 | Pharmaceutical tolerant |
| *P. veronii* IBU-12 |  | 0.0120 ± 0.003 | 0.015 ± 0.002 | Pharmaceutical tolerant |
| *P. stutzeri* IBU-13 |  | 0.103 ± 0.002 | 0.107 ± 0.002 | Pharmaceutical tolerant |
| ***R. daejeonense* IBU-14** |  | **0.011 ± 0.002** | **0.005 ± 0.002** | **Potential degrader** |
| *P. stutzeri* IBU-17 |  | 0.071 ± 0.0005 | 0.091 ± 0.001 | Pharmaceutical tolerant |
| ***R. daejeonense* IBU-18** |  | **0.012 ± 0.003** | **0.004 ± 0.001** | **Potential degrader** |
| **CBZ Enrichment** |  |  |  |  |
| ***N. carbamazepini* CBZ-1** | 0.146 ± 0.01 | **0.041 ± 0.004** | **0.021 ± 0.002** | **Potential degrader** |
| *N. carbamazepini* CBZ-2 |  | 0.004 ± 0.001 | 0.018 ± 0.008 | Growth inhibition |
| ***B. mongoliensis* CBZ-3** |  | **0.008 ± 0.001** | **0.004 ± 0.001** | **Potential degrader** |
| *P. kribbensis* CBZ-4 |  | 0.013 ± 0.001 | 0.022 ± 0.003 | Growth inhibition |
| *R. qingshengii* CBZ-6 |  | 0.012 ± 0.000 | 0.009 ± 0.000 | Pharmaceutical tolerant |
| *P. moorei* CBZ-7 |  | 0.092 ± 0.0005 | 0.063 ± 0.006 | Pharmaceutical tolerant |
| *P. veronii* CBZ-8 |  | 0.006 ± 0.001 | 0.013 ± 0.001 | Growth inhibition |
| *V. paradoxus* CBZ-9 |  | 0.004 ± 0.0005 | 0.005 ± 0.001 | Pharmaceutical tolerant |
| ***V. paradoxus* CBZ-10** |  | **0.006 ± 0.001** | **0.002 ± 0.001** | **Potential degrader** |
| *M. discipulorum* CBZ-11 |  | 0.014 ± 0.005 | 0.024 ± 0.003 | Growth Inhibition |
| *P. stutzeri* CBZ-13 |  | 0.031 ± 0.006 | 0.040 ± 0.01 | Pharmaceutical tolerant |
| *B. cereus* CBZ-16 |  | 0.0155 ± 0.006 | 0.012 ± 0.002 | Pharmaceutical tolerant |

α - STD – Standard Deviation Value

β - Non-inoculated, abiotic control samples

γ - Inoculated samples without pharmaceutical compound

δ – Inoculated samples containing one of the target pharmaceutical compounds

ε –the A610 value of the sample is notably higher than that of the blank – presumably the bacterial growth was inhibited by the presence of the tested pharmaceutical compound. The threshold for “Growth inhibition” was set to at least a 1.5-2-times difference between the obtained A610 values

θ – the A610 value of the sample is remarkably lower than that of the blank – the presence of the tested pharmaceutical compound induced a higher bacterial activity in the sample compared to the blank. The threshold for “Potential degrader” was set to at least a 2-times difference between the obtained A610 values

λ – no significant difference was detected between A610 values of blank and sample. Presumably the tested compound neither stimulated nor inhibited the bacterial growth. The isolate tolerated the presence of the tested pharmaceutical compound.
